# SUN/KASH interactions facilitate force transmission across the nuclear envelope

**DOI:** 10.1080/19491034.2019.1595313

**Published:** 2019-03-19

**Authors:** Hongyan Hao, Daniel A. Starr

**Affiliations:** Department of Molecular and Cellular Biology, University of California, Davis, CA USA

**Keywords:** LINC complex, mechanical force transduction, nuclear envelope, nuclear positioning

## Abstract

LINC complexes (Linker of Nucleoskeleton and Cytoskeleton), consisting of inner nuclear membrane SUN (Sad1, UNC-84) proteins and outer nuclear membrane KASH (Klarsicht, ANC-1, and Syne Homology) proteins, are essential for nuclear positioning, cell migration and chromosome dynamics. To test the in vivo functions of conserved interfaces revealed by crystal structures, Cain et al used a combination of Caenorhabditis elegans genetics, imaging in cultured NIH 3T3 fibroblasts, and Molecular Dynamic simulations, to study SUN-KASH interactions. Conserved aromatic residues at the -7 position of the C-termini of KASH proteins and conserved disulfide bonds in LINC complexes play important roles in force transmission across the nuclear envelope. Other properties of LINC complexes, such as the helices preceding the SUN domain, the longer coiled-coils spanning the perinuclear space and higher-order organization may also function to transmit mechanical forces generated by the cytoskeleton across the nuclear envelope.

## LINC complexes are essential for force transmission across the nuclear envelope

Eukaryotes are distinguishable from prokaryotes by the presence of a nuclear envelope separating the genetic material from the cytoplasm. The nuclear envelope is composed of two lipid bilayers and a perinuclear space continuous with the lumen of the endoplasmic reticulum (ER). Communication between the nucleoplasm and the cytoplasm is crucial for cellular functions [2]. There are three independent ways to transmit information across the nuclear envelope. Classically, small proteins and molecules can diffuse through nuclear pores while larger complexes, including ribosomal subunits, can be specifically transported through nuclear pores [3]. Alternatively, other complexes, including some virus coats, bleb from the inner nuclear membrane, diffuse across the perinuclear space in membrane-bound compartments, and fuse to the outer nuclear membrane [4]. Third, mechanical forces can be transferred directly across the nuclear envelope through conserved physical bridges termed LINC (**Li**nker of **N**ucleoskeleton and **C**ytoskeleton) complexes. Such mechanical force transmission across the nuclear envelope is essential for nuclear positioning and meiotic chromosome dynamics [5,6].

**Figure 1.**
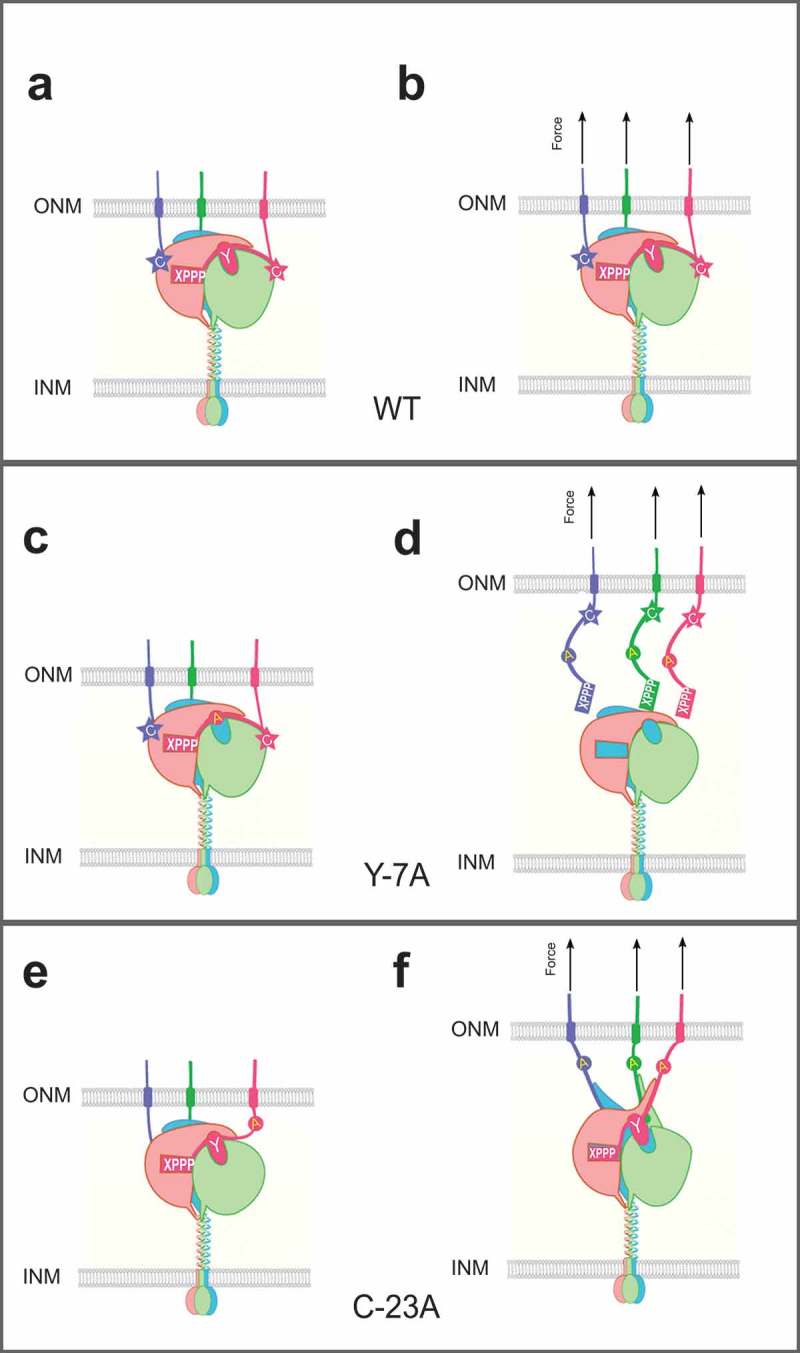
Conserved residues at SUN-KASH interfaces function in LINC complex assembly and force transmission at the nuclear envelope. Each panel shows a cartoon simulation focusing on the SUN-KASH interface. SUN proteins (salmon, light green and blue) trimerize and span the inner nuclear membrane (INM). Coiled-coils reach toward the outer nuclear membrane (ONM). A trimer of conserved SUN domains (at the top of the coils) interacts with three KASH peptides in the ONM (blue, green, and red) to form a hetero-hexamer. In wild type, the PPPX motif (rectangle) at the C terminus of KASH fits into a pocket in one SUN protomer (P1), the −7 tyrosine (oval) interacts with a hydrophobic binding site between the KASH lid of SUN P1 and the SUN core of second protomer (P2) and the −23 cysteine forms an intermolecular disulfide bond (star) with SUN. (A, C, and E) Representations of the LINC complexes without any forces. (B, D, and F) Representations of LINC complexes under mechanical strain from pulling forces generated in the cytoplasm. (A-B) wild type; when force is applied on the LINC complex, a stable LINC transfers forces across the nuclear envelope to the nucleoskeleton, resulting in nuclear movements. (C-D) KASH with a tyrosine mutation at position −7 from the C terminus; LINC complexes still form, but fail when force is applied. (E-F) KASH with a mutation in the −23 cysteine. LINC complexes form, and are able to transfer some forces across the nuclear envelope to move nuclei. However, without the intermolecular disulfide bond, LINC will break under maximal force in some systems (not shown).

LINC complexes are made of SUN (**S**ad1, **UN**C-84) proteins in the inner nuclear membrane and KASH (**K**larsicht, **A**NC-1, and **S**yne **H**omology) proteins in the outer nuclear membrane [7]. The central interaction occurs in the lumen of the nuclear envelope near the outer nuclear membrane, between a trimer of conserved SUN domains about 175 residues each and C-terminal KASH peptides of 10 to 32 residues (Figure 1) [8–12]. SUN proteins then extend across the perinuclear space, cross the inner nuclear membrane and interact with lamins or other proteins in the nucleus, including chromatin and telomeric proteins [6,13,14]. The large, cytoplasmic portions of KASH proteins associate with various components of the cytoskeleton, including actin filaments, microtubule motors, or intermediate filaments [7,15].

LINC complexes are conserved across eukaryotes, and likely played a role in the formation of the nuclear envelope before the last eukaryotic common ancestor [16]. Furthermore, LINC complexes facilitate diverse functions, ranging from pronuclear migration, skeletal muscle nuclear positioning, nuclear migration in the hair cells of the inner ear, and chromosome pairing during meiosis [5,6,17,18]. A common feature of these processes is that mechanical forces generated by the cytoskeleton need to be transferred across the nuclear envelope to the nucleoskeleton. Mutations in LINC components are linked to a wide variety of diseases including muscular diseases, hearing loss, neurological disorders, and cancers [19]. Thus, it is critical to study the molecular mechanisms for the assembly of the LINC complexes as well as how forces are transmitted through the LINC complex.

## Crystal structure of the SUN/KASH complex

A major breakthrough for understanding LINC complexes came in 2012 when the crystal structures of the interaction between human SUN and KASH domains were revealed [8,20]. Overall, three SUN domains (SUN2_540-717_) and their adjacent, short, coiled trimerization domains (SUN2_525-540_) interact with C-terminal 29 residues of Nesprin-1 or Nesprin-2 KASH domains to form a clover-like hetero-hexamer complex [8]. The SUN domain folds into a β-sandwich core with a protruding 20-residue β-hairpin (SUN2_567-587_) termed the KASH-lid. Each KASH peptide interacts with three interfaces of a SUN trimer. First, the core of a SUN2 protomer formed by hydroxyl groups of S641, Y703 and Y707, together with H628 and stabilized by the C601-C705 intermolecular disulfide bond, forms a binding pocket for the three prolines of a PPPX motif at the C terminus of the KASH peptides (Figure 1a). Second, the KASH-lid of SUN2 protomer 1 (P1) and the β-sandwich core of protomer 2 (P2) form a groove that interacts with the −4 to −14 residues of the KASH peptide. The −4, −5 and −6 residues of KASH do not interact strongly with SUN2, while a Y at −7 and a L at −9 are buried into the cleft between P1 and P2 (Figure 1a). Third, the KASH peptide kinks between the P-11 and −12 residues. Next, residues −13 to −23 of the KASH peptide bind along the surface of SUN2 P2, except for a large, conserved hydrophobic residue at −17 buried into a hydrophobic pocket. Finally, the crystal structures showed that a cysteine at −23 of the KASH peptide forms a disulfide bond with a conserved cysteine in SUN2 (C563), to further stabilize the interaction (Figure 1a) [8]. Thus, the crystal structures predict that a number of conserved residues that are likely important for the interaction between SUN and KASH proteins and their ability to transfer mechanical forces across the nuclear envelope (Figure 1b).

To test the crystal structure predictions, Sosa et al. [8], performed *in vitro* pull-down assays with SUN and KASH domains. They showed that a disulfide bond is formed between SUN and KASH proteins in HeLa cells. However, a truncated KASH peptide of only 14 amino acids still interacts with SUN proteins *in vitro*, indicating that the cysteine at −23 and the disulfide bond between SUN and KASH are not required for their interaction. In contrast, extending the C terminus of the KASH peptide by a single alanine residue, or mutating conserved residues at −7 or −9 of KASH peptides abolishes SUN-KASH interactions *in vitro* [8]. The *in vivo* functional significance of the different SUN-KASH interaction interfaces and the implications for LINC complex mechanisms were yet to be uncovered. It was hypothesized that strong interactions between SUN and KASH domains are required to resist forces generated from the cytoskeleton and to ensure effective force transmission across the nuclear envelope. We set out to test this model in live cells.

## Conserved residues are required for functional LINC complex in live cells

In our recent study [1], we tested the ability of conserved residues for SUN-KASH interactions and force transmission. To examine these processes in cells, we used *C. elegans* developing hypodermal tissues. During *C. elegans* embryogenesis, hypodermal precursor nuclei migrate to the opposite side of the cell in a SUN-KASH dependent manner [21]. The SUN protein UNC-84 recruits the KASH protein UNC-83 to the outer nuclear membrane [22]. UNC-83 then serves as the cargo adaptor to recruit microtubule motors kinesin-1 and dynein to the surface of nuclei [7,23,24]. The motors facilitate nuclear migration towards the plus end of microtubules [24]. Failure of this nuclear migration leads to ectopic localization of nuclei in the dorsal cord of the L1 larva [25]. After nuclear migration, hypodermal cells fuse to form a syncytium and nuclei are anchored in place. In adult *C. elegans* more than a hundred nuclei are evenly spaced in the hyp7 syncytium [26]. The KASH protein ANC-1 interacts with the SUN protein UNC-84 to anchor nuclei to the actin cytoskeleton. In *unc-84* and *anc-1* null mutants, hyp7 nuclei form clusters [27,28].

We used a mammalian tissue culture model to complement our *C. elegans* studies. NIH3T3 fibroblasts cultured in a monolayer polarize with centrosomes in front of nuclei in response to a scratch wound [29]. To achieve this polarization, nuclei move rearward, away from the wound edge [30]. The forces to move nuclei arise by tethering nuclei to rearward-moving actin cables. During migration, the N-terminal calponin homology (CH) domains of Nesprin-2G bind actin while its KASH domain connects to SUN2 in the nuclear envelope to form a LINC complex [31]. The LINC complex is under tension in this process, indicated by a fluorescence resonance energy transfer (FRET)-based tension biosensor [32]. A mini-Nesprin-2G (EGFP-mini-N2G) construct containing CH domains and the KASH domain is sufficient for the nuclear movement in polarizing NIH3T3 fibroblasts [31]. Interestingly, the luminal domains of KASH proteins differ in length; Nesprin-2G and ANC-1 contain a long KASH domain that harbors the −23 conserved cysteine while the UNC-83 KASH domain only contains 18 residues and is missing the conserved cysteine.

To test the function of conserved residues of SUN-KASH interfaces in live cells, our collaborators, Natalie Cain and Gant Luxton, led teams that tested predictions from the crystal structure and *in vitro* assays in the context of nuclear positioning in developing *C. elegans* hypodermal cells and polarizing NIH 3T3 fibroblasts, respectively [1]. We first showed that extending the C terminus of the UNC-83 KASH domain by a single alanine completely disrupts nuclear migration during *C. elegans* embryonic hypodermal development. Moreover, the SUN-KASH interaction is likely abolished since UNC-83(KASH+A) was undetectable on the nuclear envelope by immunostaining. These results fit the crystal structure and the *in vitro* pull-down data [8]. However, not all of our findings matched the crystal structural predictions. Mutating the −7 tyrosine of the UNC-83 KASH domain did not completely block the SUN-KASH interaction (Figure 1c). UNC-83(Y967A) still localized to the nuclear envelope prior to nuclear migration, suggesting that it was able to at least weakly interact with UNC-84. However, nuclear migration failed, indicating that the UNC-83(Y967A) mutant could not interact with UNC-84 SUN domains strongly enough to transfer the mechanical forces needed to move nuclei across the nuclear envelope (Figure 1d). Since the UNC-83 KASH domain is normally only 18 amino acids in length and the 18-mer KASH peptide used in the pulldown assay to introduce the Y-7A mutation also lacks the conserved −23 cysteine, it remained unclear how the mutation at the −7 aromatic residue could be tolerated in long KASH domains with intermolecular disulfide bonds between SUN and KASH. We developed nuclear positioning assays in adult *C. elegans* hypodermal syncytia as a model to test the function of longer KASH domains [25]. Interestingly, mutating the phenylalanine residue at −7 of the ANC-1 KASH domain did not cause nuclear anchorage defects [1]. This indicates that nuclear anchorage in *C. elegans* hyp7 syncytia might not require as high of forces to be transmitted through LINC complexes as nuclear migration does. In contrast, mutating the Nesprin-2G KASH tyrosine at −7 to an alanine disrupted rearward nuclear movement in polarizing NIH 3T3 fibroblasts, indicating that Y-7 is important for force transmission across the SUN2/Nesprin-2 LINC complex (Figure 1d). Thus, the −7 conserved aromatic residue is important for LINC complexes under mechanical tension, but not required for initial interactions between SUN and KASH domains.

## Conserved cysteines regulate LINC complex function during nuclear positioning

The longer KASH domains of *C. elegans* ANC-1 and mammalian Nesprin-2 harbor a conserved cysteine at position −23, which forms an intermolecular disulfide bond with SUN trimers in the crystal structures. However, the shorter KASH domain of *C. elegans* UNC-83 does not contain the interface with P2 or the −23 cysteine. We, therefore, tested the role of the −23 cysteine in nuclear anchorage in *C. elegans* and nuclear rearward movement in polarizing NIH 3T3 fibroblasts. In support of our model, mutating the −23 cysteine in ANC-1, Nesprin-2, or their partner cysteines in UNC-84 or SUN2, disrupted nuclear positioning [1]. Since the cysteine is not required for the SUN/KASH interaction in the in vitro pulldown assay [8], we proposed that the nuclear positioning defects might be caused by insufficient force transmission across the LINC complex (Figure 1e-f). To further test our hypothesis that a disulfide bridge is needed for maximal force transduction across LINC complexes, our collaborators Zeinab Jahed and Mohammad Mofrad used Molecular Dynamic simulations to apply a constant velocity of 0.05m/s pulling on the N termini of KASH peptides associated with the wild type or C953A SUN2 trimer. When an intermolecular disulfide bond was present, the pulling forces were transmitted effectively through the LINC complex to the coiled-coil regions (Figure 1b). However, in the cysteine mutant, the SUN/KASH interactions were not strong enough to withstand the forces and the KASH peptide and KASH lid of the SUN protein underwent significant stretching (Figure 1f) [1]. Taken together, our cellular and modeling data support the hypothesis that an intermolecular disulfide bridge is needed to stabilize SUN-KASH interactions to transmit maximal forces through LINC complexes.

Many other KASH domains, including mammalian KASH5 and *C. elegans* UNC-83 and ZYG-12, contain short KASH domains without the conserved cysteine. All three of these proteins function during brief windows in development to move nuclei and/or chromosomes inside meiotic prophase nuclei. In our assay, UNC-83-mediated nuclear migration was not affected in *unc-84(C953A)* mutants. Thus, short KASH domains can interact with SUN domains in the absence of disulfide bonds and can transmit enough force to move nuclei or chromosomes. Interestingly, the *anc-1(CC-23, −24-AA)* mutation caused nuclear anchorage defects while the *anc-1(F-7A)* mutant was nearly wild type [1]. Further molecular dynamic simulations or *in vivo* assays are required to investigate force transmission across LINC complexes formed by longer or shorter KASH domains, with or without the disulfide bond, and with or without the −7 aromatic amino acid. In addition, it could be valuable to use FRET tension biosensors to determine the amplitude and direction of force across the LINC complex during different processes [32].

## Other factors that influence force transmission through LINC complex

Besides the strong interaction between SUN and KASH domains, four other properties of LINC complexes may be important for force transmission across the nuclear envelope. First, helical domains of SUN proteins are predicted to regulate when SUN domains interact with KASH proteins to turn LINC complexes on and off. Second, the predicted extended coiled-coils in SUN proteins transmit forces by spanning the perinuclear space. Third, SUN and KASH domains could directly interact with the inner nuclear membrane. Finally, coiled-coils and other domains in LINC complexes appear to contribute to higher order oligomerization of LINC complexes. Together, these properties of LINC complexes would maximize the transfer of mechanical forces from the cytoskeleton to the nucleoskeleton. Further studies of these mechanisms are required to fully understand how LINC complexes function.

The timing of SUN protein activation and LINC complex assembly must be regulated. Structural and biochemical studies revealed that the helical region closest to the SUN domain serves as the minimal trimerization domain [8,33]. Moreover, recent crystal structures identified a nearby short alpha-helical bundle that is able to engage with a single SUN domain through the KASH-lid, locking the protein in a monomeric, presumably inactive state [34,35]. Mutating residues at the interface between this alpha helix and the KASH-lid restored the KASH-binding capacity of the SUN domain [35]. Interestingly, the KASH-lid is released from the autoinhibited state in the presence of the trimerization coiled-coil in Molecular Dynamic simulations [36]. Both the crystal model and simulations indicate that a calcium ion can occupy a cation loop formed in the extended coiled-coil domain of the SUN protein, which might play a role in activating the LINC complex. When a conserved residue (E452) in the mouse SUN2 trimerization coil is not occupied by Ca^2+^, it interacts with the SUN domain and potentially inactivates LINC complex formation. Both the E452 mutation in SUN2 and Ca^2+^ concentration changes monomer to trimer ratios in gel filtration assays [36]. Notably, the E452D mutation in human SUN2 was identified in muscular dystrophy patients [34–36]. Further *in vivo* studies are needed to demonstrate the significance and mechanisms for the SUN protein activation, which is essential to form LINC complex and transfer force across the nuclear envelope.

A second mechanism influencing force transmission involves predicted coiled-coils in SUN proteins. These coiled-coils are thought to span the perinuclear space and connect SUN domains near the outer nuclear membrane to the inner nuclear membrane. Coiled-coil domains are elastic structures often found in cytoskeletal proteins such as kinesin, myosin and vimentin intermediate filaments [37,38]. How SUN proteins span the perinuclear space is not clear at a structural level. Mammalian germ cell-specific SUN proteins, SUN3-SUN5, contain shorter coiled-coil domains, which is predicted to bring the two nuclear envelope membranes closer together [39]. However, shortening the coiled-coil domains of UNC-84 did not shrink the perinuclear space and had little effect on UNC-83-mediated nuclear migration in *C. elegans* [40]. It will be interesting to further test the role of the coiled-coils in force transmission across LINC complexes in other processes and to determine more complete structures of LINC complexes.

The third potential aspect of force transmission across the nuclear envelope is how LINC components could directly interact with the lipid bilayer or chromatin to further stabilize the complex. How LINC complexes interact with lipid membranes is poorly understood. For example, *in vitro* reconstituted LINC complexes on artificial nuclear membranes indicate that SUN2 contains a single transmembrane domain while SUN1 has three [41]. In addition, hydrophobic patches in the nucleoplasmic region of SUN proteins may associate with the inner nuclear membrane and the lamin network to distribute forces inside the nucleus [41]. Additional studies are required to determine how SUN and KASH domains might be directly interacting with the outer nuclear membrane. Crystal structures showed that a conserved hydrophobic patch exists on the surface of the SUN domain at a position facing the outer nuclear membrane, suggesting that SUN domains are extending into the outer nuclear membrane lipid bilayer when binding KASH [8,20,34]. Finally, the interaction between KASH proteins and the cytoplasmic leaflet of the outer nuclear membrane is potentially stabilized to maximize force transmission. Recently, an outer nuclear membrane protein Kuduk was shown to interact with LINC complexes in the outer nuclear membrane in *Drosophila*. Kuduk (TMEM258 in mammals) has a cytoplasmic amphipathic helix as well as a trans-membrane helix that both interact with the outer nuclear membrane and stabilize LINC [42]. Future experiments should uncover how Kuduk/TMEM258 might function to stabilize the LINC complexes *in vivo*.

A fourth mechanism that may allow LINC complexes to maximize the force transfer across the nuclear envelope might involve assembly into higher-order structures. There are many examples of LINC complexes organizing in larger groups. In *C. elegans*, immunostaining of UNC-84 and UNC-83 shows punctate distribution of LINC complexes on the nuclear envelope during nuclear migration [21,22]. SUN1 punctate are also observed in Hela cells [43–45]. Another case for higher-order organization of LINC complex involves how nuclei move rearward in wounded NIH3T3 fibroblasts. During nuclear migration, linear arrays of SUN2-Nesprin-2G LINC complexes, termed transmembrane actin-associated nuclear (TAN) lines, assemble to tether the nuclei to moving actin cables [9,31]. As a more extreme example, LINC complex proteins involved in meiotic chromosome dynamics and telomere bouquet formation redistribute and concentrate in large patches associated with chromosome ends [6,46–48].

Recent studies suggest possible mechanisms for LINC complexes to assemble into larger structures., Mammalian SUN1 proteins form oligomers larger than trimers detected in living cells by fluorescence fluctuation spectroscopy [49]. SUN1 has also been observed to form clusters in a reconstituted, cell-free expression system on artificial lipid bilayers [41]. Computer modeling predicts that SUN1 trimers can associate with each other laterally through their SUN domains to form higher-order clusters [41,43]. It has also been proposed that a cysteine adjacent to the SUN domain of SUN1 protein is able to form intermolecular disulfide bonds with neighboring SUN trimers [44,50]. The specific requirement of mammalian SUN1, but not SUN2, during meiotic chromosome pairing, might be associated with the ability of SUN1 to more easily form higher-order structures than SUN2 [9,43,44,49]. Interestingly, a recent study has shown different mechanisms involved in homeostatic nuclear positioning under centrifugal force. Rearward nuclear recentration requires TAN lines formed by SUN2 and Nesprin-2G while the same KASH protein Nesprin-2G interacts with SUN1 and the microtubule motor dynein to facilitate forward recentration [51]. It is not clear if distinct higher-order structures are specialized for force transmission or if oligomerization states of SUN protein are involved in selection between SUN1 and SUN2 by Nesprin-2G in different nuclear positioning processes. Further studies are required to determine the physiological significance and molecular mechanism of higher-order LINC organization and force transmission.
